# Three-Dimensional Human Neurovascular Unit Modeling Reveals Cell-Specific Mechanisms of Traumatic Brain Injury

**DOI:** 10.3390/jfb16120454

**Published:** 2025-12-07

**Authors:** Liam H. Power, Evan C. Marcet, Zihong Chen, Jinpeng Chen, Artem Arkhangelskiy, Michael J. Whalen, Ying Chen, David L. Kaplan

**Affiliations:** 1 Department of Biomedical Engineering, Tufts University, Medford, MA 02155, USA; liam.power@tufts.edu (L.H.P.); evan.marcet@tufts.edu (E.C.M.); jinpeng.chen@tufts.edu (J.C.); artem.arkhangelskiy@tufts.edu (A.A.); 2 Department of Pediatrics, Massachusetts General Hospital and Harvard Medical School, Boston, MA 02114, USA; mwhalen@mgh.harvard.edu

**Keywords:** cellular responses, traumatic brain injury, neurovascular unit dysfunction

## Abstract

Severe traumatic brain injury includes neurovascular unit (NVU) damage that is linked to the later development of neurodegenerative diseases. Cell-type-specific contributions and crosstalk between cells of the neurovascular unit following brain injury remain poorly defined in human cells. Here, we developed a three-dimensional (3D) human NVU model using silk–collagen scaffolds to examine cellular responses to controlled cortical impact (CCI). Using this platform, we show that CCI induced acute cell death in astrocytes, microglia, and endothelial cells but spared pericytes, which occurred independently of classical apoptotic or necroptotic pathways. Astrocytes and microglia were the primary sources of early bioactive IL-1β release, while endothelial junctional integrity was differentially regulated by support cells: astrocytes destabilized VE-cadherin, pericytes preserved barrier proteins, and microglia contributed to Claudin-5 loss in multicellular settings. Conditioned media experiments demonstrated that soluble factors from injured support cells alone were sufficient to disrupt endothelial junctional proteins (ZO-1 and Occludin) and induce inflammatory adhesion molecules (ICAM-1 and VCAM-1). Together, these findings define cell-type-specific injury responses and reveal how NVU interactions regulate vascular dysfunction after trauma, providing a human-based framework for understanding blood–brain barrier (BBB) disruption following traumatic brain injury (TBI).

## 1. Introduction

Traumatic brain injury (TBI) is a leading cause of death and disability among young people. TBI injuries are a risk factor for developing chronic neurologic deficits later in life [1,2,3]. The mechanisms that link TBI and the development of chronic cognitive impairment are not yet defined, though emerging evidence suggests neurovascular unit (NVU) dysfunction after TBI is a contributing factor to chronic disease states, including Alzheimer’s disease (AD), Parkinson’s disease (PD), and amyotrophic lateral sclerosis (ALS) [4,5,6,7]. The neurovasculature is lined by brain microvascular endothelial cells and supported by pericytes, astrocytes, and nearby microglia that together regulate nutrient exchange and protect the brain [8]. The endothelium of the NVU is defined by the expression of junctional proteins that restrict paracellular transport and reduced levels of endocytic vesicles to limit transcellular passage through the BBB [8,9,10]. Though inflammation is important to remove damaged cells and initiate repair, prolonged or dysregulated inflammation may contribute to neuronal damage [5,6,11]. Strategies to protect the neurovascular unit after TBI require a more complete understanding of injury dynamics and cell–cell interactions in the NVU (pericytes, astrocytes, and microglia), as well as cell-specific molecular processes that may drive injury responses.

Existing strategies to isolate cell-specific contributions to NVU dysfunction rely heavily on animal models, which frequently fail to predict or recapitulate human outcomes due to species-specific variations (anatomic differences, gene expression differences, or protein function differences) [12]. Post-mortem human tissue offers clues to pathogenesis and mechanisms underpinning disease states in humans, yet tissue samples are acquired post-mortem, often months or years after the initial insult, and provide little insight to cell states and stresses in the immediate or subacute period of injury [13,14]. Moreover, in vivo models are difficult to parse at the cellular level, since systemic responses (e.g., inflammation, circulating factors, or compensatory repair mechanisms) often obscure the direct contributions of individual NVU cell types. In contrast, human-based in vitro models permit direct manipulation of specific cell types.

To bridge these gaps, we employ mechanically injured human in vitro tissue models to study TBI at the cellular level. Our platform utilizes controlled cortical impact (CCI)—a method well-established in murine TBI studies and adapted for 3D neuronal cultures [15]—to induce trauma in customizable, human cell-based NVU models, which builds on our previous work [16]. These models are constructed using 3D composite silk–collagen scaffolds seeded with human primary brain microvascular endothelial cells (BMECs), astrocytes, pericytes, and microglia in mono-, co-, or triculture configurations, allowing systematic dissection of cell-type-specific injury responses. In vitro modeling of TBI has become increasingly popular due to the ability to control injury parameters and cell-specific responses [17]. Although there has been a push to model neuronal response in 3D in vitro models [18,19], complex NVU models have only recently gained traction in the field [20,21]. To add novelty beyond characterizing differential cellular reactions to mechanical trauma, we investigate the roles of necroptosis, apoptosis, and non-programmed necrosis in initial injury progression. This approach serves to clarify the complex cell-type-dependent relationship between cell death and neuroinflammation and offers new mechanistic insights into TBI pathology that remain unexplored. We hypothesized that NVU support cells would differentially influence endothelial responses following CCI through both direct contact and paracrine signaling, thereby shaping acute injury outcomes.

## 2. Methods

Experimental design—Human neurovascular unit (NVU) tissue models were constructed using 3D silk scaffolds seeded with primary human brain microvascular endothelial cells (hBMECs), astrocytes, pericytes, and microglia in mono-, co-, or triculture configurations. After a 7-day culture period, scaffolds were subjected to controlled cortical impact (CCI) or sham treatment to model traumatic brain injury. Cell death, cytokine release, and junctional protein expression were quantified 24 h post-injury. Pharmacologic inhibitors of apoptosis (zVAD-fmk) and necroptosis (Nec-1) were used to evaluate pathway dependence. To assess paracrine signaling, media collected from injured monocultures were applied to confluent endothelial monolayers for 72 h, after which markers of barrier integrity (ZO-1, Occludin, Claudin-5, and VE-cadherin) and inflammation (VCAM-1, ICAM-1, and IL-1β) were analyzed. This design enabled identification of cell-type-specific injury responses and elucidation of direct versus soluble-factor-mediated effects on NVU health and BBB integrity following mechanical trauma and downstream inflammatory signaling.

Fabrication of 3D silk scaffolds—A solution of 6% silk fibroin was prepared from *Bombyx mori* (*B. mori*) silkworm cocoons, as in prior studies. A 30 mL of the silk solution was gently poured into a 10 cm dish and incubated with 425–500 µm sodium chloride (NaCl) particles. After 2 days of incubation, the mixture was incubated for 1 h at 65 °C to induce beta-sheet formation, forming a 3D sponge. The sponge was removed from the dish and subjected to 2 days of dialysis at room temperature to remove all of the sodium chloride. Scaffolds were cut with 6 mm biopsy punches (McMaster-Carr, Princeton, NJ, USA) and trimmed with a razor blade (Fisher Scientific, Hampton, NH, USA) to a height of 2 mm. Prior to seeding, scaffolds were sterilized using an autoclave (20 min) and stored at 4 °C before use.

Cell Culture and Seeding of Scaffolds—Primary human brain microvascular endothelial cells (BMECs) were purchased from Cell Systems (Cat #: ACBRI376, Kirkland, WA, USA) and AngioProteomie (Cat #: cAP-0002GFP, Boston, MA, USA). Human brain pericytes (Cat #: 1200) and human astrocytes (Cat #1800) were purchased from ScienCell (Carlsbad, CA, USA), and RFP-Pericytes were purchased from AngioProteomie (Cat #: cAP-0030RFP). All cells were cultured according to the manufacturer’s protocol. When culturing endothelial cells, pericytes, and astrocytes together, a 1:1 mixture of endothelial cell media (Cat #: cAP-02, AngioProteomie) and astrocyte media (Cat #: 1801, ScienCell) was used for culture (referred to as “tri-culture media”). For this entire study, primary cells from passage numbers 3–7 were used for seeding in 3D. All silk scaffolds were moved into 48-well plates (Corning, Corning, NY, USA) and coated with 50 µg/mL human fibronectin (Sigma-Aldrich, St. Louis, MO, USA) for 1 h at 37 °C before cell seeding. The coating solution was removed via vacuum aspiration. A concentrated cell mixture of 2.5  ×  10^5^ BMECs alone (endothelial monocultures) (5 × 10^6^ cells/mL) or 2.5  ×  10^5^ BMECs, 1.25  ×  10^5^ pericytes, and 1.25  ×  10^5^ astrocytes (tricultures, 10 × 10^6^ cells/mL) was seeded into each scaffold in 50 µL of media via pipette. For mono- or co-cultures containing microglia, a consistent seeding density of 1.25  ×  10^5^ microglia per scaffold was used. Each scaffold was gently squeezed against the well wall with a pipette tip to allow the silk sponges to fully absorb the cell suspension. Well plates were then stored in the incubator for 1 h to allow cell attachment, prior to adding 1 mL of media to each well to fully cover the scaffolds (1 mL). Cell-seeded sponges were cultured for 24 h before being carefully moved into 96-well plate wells (Corning) and coated with 100 µL of ECM proteins. Collagen type I gels were prepared from rat tail type I collagen (Cat #: 5153, Advanced Biomatrix, Carlsbad, CA, USA), triculture media, and 1 N NaOH at a final concentration of 3 mg/mL for experimental treatments and statistical testing. NVU 3D models were cultured for at least 7 days with media changes every three days. For these experiments, E refers to endothelial cells alone, A refers to astrocytes, P refers to pericytes, and M refers to microglia. In co- or tricultures, cells were seeded according to ratios established previously and labeled according to the cells included in the given scaffold. For all co- and triculture conditions, the indicated cell types were seeded simultaneously on the same 3D scaffold to allow direct physical contact and cell-to-cell interactions. Prior to treatment, cells were maintained in the “triculture media” established previously. For treatment and analysis of cytokine release, media were switched to EGM^®^2 MV Microvascular Endothelial Cell Growth Medium-2 BulletKit (0.1% FBS, including IGF-1, hydrocortisone, EGF, and Gentamicin/Amphotericin).

Traumatic Injury—Three-dimensional models were placed on a flat weigh boat and injured using a Controlled Cortical Impact (CCI) device with a pneumatic cylinder with the following parameters: velocity: 6 m/s, penetration depth: 0.6 mm, dwell time duration: 200 ms. SHAM injured tissues were handled in the same way, but without injury. These parameters are in line with our previous works and are identical to standard CCI injuries performed in vivo using rodent models [15,22].

Drug Treatment—Three-dimensional triculture tissue models were pretreated prior to mechanical impact with pan-caspase inhibitor Z-Val-Ala-Asp-(Ome)-fluoromethylketone (zVAD-fmk, 30 µM, ToCris 2163) or 5-(Indol-3-ylmethyl)-(2-thio-3-methyl)hydantoin (Necrostatin-1, 30 µM, Sigma 480065) spiked into triculture media for 30 min. Use of zVAD-fmk inhibits all caspases, including those relevant for inflammasome activation and IL-beta release (caspase 1 and caspase 4/5), as well those related to apoptosis and necroptosis (caspase 3 and caspase 8). Necrostatin-1 (Nec-1) is a small molecule inhibitor of receptor-interacting serine/threonine-protein kinase 1 (RIPK1).

Media Transfers—To assess the impact of brain-specific supporting cell paracrine signaling 24 h after CCI in 3D monocultures of microglia, astrocytes, endothelial cells, and pericytes, media from these wells were transferred to a post-confluent brain microvascular endothelial monolayer for 72 h. After 72 h treatment, cells from these monolayers were collected for protein and mRNA isolation.

Western Blot—Scaffolds frozen at −80 °C were placed in a solution of 1X RIPA (Sigma, 20-188) and protease and phosphatase inhibitor cocktail (Invitrogen, Carlsbad, CA, USA, A32961) and sonicated with 20 pulses at 20% amplitude. Bradford assay (ThermoFisher, 23238) was used to quantify protein concentration in the lysate. Then, 15–20 ug of protein was loaded into 4–12% Bolt Bis-Tris gels (ThermoFisher, NW04125BOX) and transferred using an iBlot2 Transfer system onto nitrocellulose membranes (ThermoFisher, IB23002) at 23 V for 4 min. Membranes were blocked with 5% BSA (Jackson Immuno Research Labs, West Grove, PA, USA, 001-000-161) in TBST (Boston BioProducts, Ashland, MA, USA, IBB-181) and then incubated with primary antibodies in 5% BSA overnight at 4 °C on a rocker. Primary Antibodies used were human anti-platelet endothelial cell adhesion molecule (anti-PECAM1/CD31, Abcam ab9498, 1:1000), anti-Claudin 5 (anti-CLDN5, Abcam ab15106, 1:1000), anti-zonula occludens-1 (anti-ZO-1, Thermo Fisher, 33-9100, 1:500), anti-vascular endothelial cadherin (anti-VE-cadherin, CST, 2500S, 1:1000), anti-nuclear factor kappa-light-chain-enhancer of activated B cells (anti-NFκB, CST, 8242S, 1:1000), anti-phospho-nuclear factor kappa-light-chain-enhancer of activated B cells (anti-p-NFκB, Ser536, CST, 3033S, 1:1000), anti-IL-1β (Abcam, ab9722, 1:1000), ant-CD54/ICAM-1 (CST, 26104, 1:1000), anti-VCAM-1 (CST, 13662, 1:1000), anti-RIPK1 (BD Biosciences, San Jose, CA, USA, 610459, 1:1000), anti-Caspase-1 (CST, 2225, 1:1000), anti-Occludin (CST, 91131, 1:1000), and anti-VEGFA (CST, 50661, 1:1000).

The following day, membranes were washed in TBST 3x for 5 min before adding fluorescent secondary antibodies diluted in 5% BSA at a 1:30,000 ratio for 1 h on a shaker (Cell Signaling Technology, Danvers, MA, USA, 5257P and 5151P). The membranes were then imaged using a BioRad Chemidoc MP on the Dylight 800 fluorescent setting. β-actin levels were detected with a primary antibody conjugated to HRP (Abcam, ab49900, 1:10,000) using a BioRad Chemidoc MP on the chemiluminescent setting after incubating the blot for 5 min in West Femto-enhanced chemiluminescent (ECL) HRP substrate (ThermoFisher, 34096).

Lactate Dehydrogenase Activity Assay—Cellular viability of 3D BMEC cultures was measured with a lactate dehydrogenase assay (LDH) (MAK066-1KT, Sigma) following the manufacturer’s protocol. Viability was measured by collecting media released from scaffolds over the course of 24 h at 1, 2, and 3 weeks after seeding and frozen at −80 °C for storage until the assay was performed. Next, 50 μL of the media was used per assay reaction, and the amount of NADH was measured at 450 nm using a Varioskan LUX plate reader. Initial absorbance measurements were compared to the calibration curve of each plate to determine the amount of NADH present in each well, and LDH activity was subsequently calculated based on the time of the reaction and the dilution of the samples.

Phospho-RIP (Ser166) ELISA—Activated RIPK1 kinase (phospho-RIPK1) was detected with a PathScan^®^ RP Phospho-RIP (Ser166) Chemiluminescent Sandwich ELISA Kit (Cell Signaling Technologies, Catalog #: 88918) according to the manufacturer’s instructions from 3D cell scaffolds lysed in 1X lysis buffer with protease phosphatase inhibitor cocktail, sonicated on ice, and with supernatant isolated following centrifugation.

Total Human IL-1β ELISA—Total IL-1β found in the media was detected with a Total Human IL-1β High Sensitivity ELISA Kit (Invitrogen, Catalog #: BMS224-2HS) according to the manufacturer’s instructions. IL-1β levels were corrected to account for IL-1β levels found in the media alone and calculated according to results from a standard curve.

HEK Blue Culture and Bioactive IL-1β Assay—HEK-Blue IL-1β cells (Cat# hkb-il1bv2, Invivogen, San Diego, CA, USA) were cultured in high glucose DMEM (Cat# 11965092, Gibco, Waltham, MA, USA), supplemented with 10% heat-inactivated fetal bovine serum (HI-FBS; Cat# A5256801, Gibco) and 1% antibiotic–antimycotic (Cat# 15240062, Gibco), according to the manufacturer’s protocol. Briefly, HEK-Blue IL-1β cells were allowed to reach 80% confluency in a T-175 flask before dissociation with PBS for 4 min at 37 °C. After centrifugation at 150× *g* for 10 min, 50 µL/well of test media collected from any cell type was added to a 96-well plate. The HEK-Blue IL-1β cell pellet was resuspended in growth media and added at 5 × 10^4^ cells/well in 150 µL/well of media. Cells were allowed to adhere overnight, and cleaved, bioactive IL-1β was allowed to bind to IL-1R, which was genetically engineered in HEK-Blue IL-1β cells to express secreted embryonic alkaline phosphatase (SEAP), in which an increase correlates with an increase in bioactive IL-1β. On day two, 20 µL of SEAP-containing media was added to 180 µL of QUANTI-Blue Solution (Cat# rep-qbs, Invivogen) to stain SEAP. The colorimetric assay was analyzed at an absorbance wavelength of 630 nm using a spectrophotometer, with higher absorbance values indicating an increase in bioactive IL-1β.

Cytokine Arrays—Sample media were collected 24 h after injury and cytokines were measured by means of chemiluminescence using a commercial array, Human Cytokine Array C3 (RayBiotech Inc., Norcross, GA, USA), following the manufacturer’s instructions. Blot intensities were measured using a BioRad Chemidoc MP (Portland, ME, USA) and blot intensities were all normalized to each other using ImageJ2 v1.54p. To simplify cytokine array data, principal component analysis (PCA) was used to visualize and compare overall cytokine profiles to yield distinct clusters according to cell type and treatment conditions. Furthermore, one-way ANOVA (Tukey–Kramer) was run across all 40 cytokines to identify differentially expressed cytokines. Principal component analysis and biplots were calculated in Prism, and heatmaps and hierarchical clusters were generated in Rstudio using the pheatmap and ggplot2 packages.

Quantitative RT-PCR—Total RNA was isolated from human BMEC and NVU cultures in scaffolds using the RNeasy Mini Kit (Qiagen, Germantown, MD, USA). Samples were collected on day 2, day 7, and day 14 after seeding and collagen embedding. Complementary DNA was generated using iScript (Bio-Rad, Hercules, CA, USA) according to the manufacturer’s protocols. Quantitative reverse transcription PCR (qRT-PCR) was performed using Taqman Fast Advanced Master Mix and the CFX96 Real-Time PCR Detection System (Bio-Rad) and normalized against the housekeeping gene glyceraldehyde phosphate dehydrogenase (GAPDH) and then platelet endothelial cell adhesion molecule (PECAM-1/CD31). Taqman primers (Catalog # 4331182): CD-31/PECAM-1 (Assay ID: Hs01065279_m1), GAPDH (Assay ID: Hs00533949_s1), Claudin-5 (Assay ID: Hs00533949_s1), VE-Cadherin (Assay ID: Hs00901465_m1), ZO-1 (Assay ID: Hs01551861_m1), Laminin-a4 (Assay ID: Hs00935293_m1), and Collagen Type IV (Assay ID: Hs00266237_m1).

Statistical Analysis—Data are presented in figures as the mean ± SEM (*n* = 3–5) and were analyzed using Graphpad PRISM 10 (La Jolla, CA, USA). Comparisons between groups are presented as differences in means ± SEM, adjusted *p*-value. A two-tailed *t*-test was performed to compare means between two groups, whereas analysis of variance (ANOVA) was performed to compare the means of multiple groups. An adjusted *p*-value less than 0.05 was considered statistically significant.

## 3. Results and Discussion

After 7 days of 3D culture, scaffolds with NVU cells were subjected to CCI using parameters matching murine TBI studies [15,23]. This severe contusion injury induced significant cell death across all NVU configurations. This setup enabled a systematic evaluation of cell-type-specific and interactive responses within the NVU. A visual representation of 3D silk–collagen scaffold colonization by endothelial cells, pericytes, and astrocytes, obtained using SEM and confocal micrographs, is provided in Supplementary Figure S1.

### 3.1. Acute Cell-Type-Specific Cell Death and IL-1β Responses

**Figure 1:**
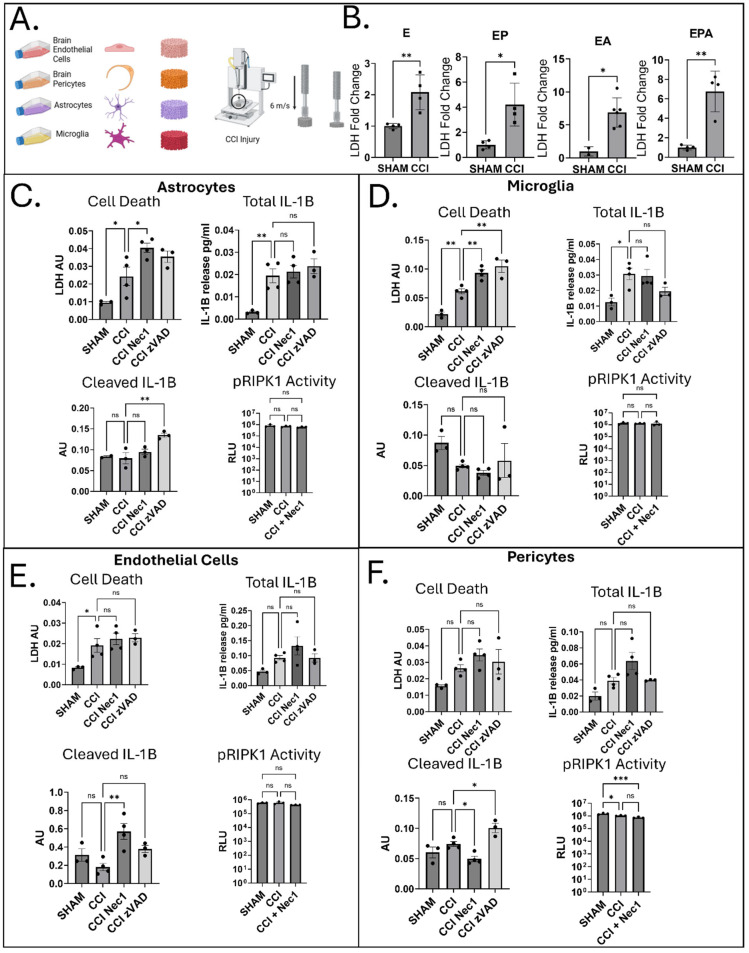
Cell Death and Cytokine Responses to TBI. (**A**) Schematic of seeding primary brain microvascular endothelial cells, pericytes, astrocytes, and HMC3 microglia into porous silk scaffolds, followed by mechanical trauma. (**B**) Multicell-type scaffolds show an increase in extracellular release of LDH into the media at 24 h after injury. Differences between SHAM/CCI compared with Student’s *t*-test. Data are presented as the mean ± SEM. *n* = 4 per group, *p* < 0.01. (**C**–**F**) Comparison of individual monoculture scaffold response to mechanical trauma with the apoptosis inhibitor zVAD, necroptosis inhibitor Nec-1 for total cell death (LDH), total IL-1β, cleaved IL-1β, and pRIPK1 activity. Data are presented as the mean ± SEM. *n* = 3 per group. Differences among media released from injured scaffolds were estimated using one-way ANOVA. Comparisons for each pair were carried out by employing Tukey–Kramer. ns, not significant (*p* > 0.05); * *p* < 0.05; ** *p* < 0.01; *** *p* < 0.001. Figure 1A was created with BioRender 2025 (Tufts University institutional license).

To assess acute cell-type-specific responses to CCI, cell death and IL-1β were assayed in all NVU co-culture combinations. LDH release, a marker of membrane damage [24], was markedly elevated in endothelial monocultures (*p =* 0.0084), endothelial–pericyte cocultures (*p =* 0.0101), endothelial–astrocyte co-cultures (*p =* 0.0121), and endothelial–astrocyte–pericyte tricultures (*p =* 0.0015) compared to sham controls (Figure 1B).

To elucidate cell-specific vulnerability to cell death after CCI, we then measured LDH release in 3D monoculture scaffolds for each of the four brain cell types. At 24 h post-injury, brain cell types showed distinct, cell-specific responses to trauma. Injured astrocytes, microglia, and endothelial cells all exhibited significantly increased LDH release compared to uninjured controls (astrocytes: *p* = 0.0480; microglia: *p* = 0.0036; endothelial cells: *p* = 0.0452), indicating loss of membrane integrity and subsequently cell death. In contrast, pericytes did not show significant LDH elevation (Figure 1C–F).

To investigate the underlying mechanisms of CCI-induced cell death, we tested two pharmacological inhibitors: Necrostatin-1 (Nec-1), which blocks necroptosis by targeting RIPK1 kinase activity [25], and zVAD, a pan-caspase inhibitor that suppresses caspase-mediated apoptosis, including pathways dependent on Caspase-8 [26]. Notably, neither Nec-1 nor zVAD reduced LDH release in any cell type, suggesting that the cell death observed was not driven by classical apoptosis or necroptosis. Because Caspase-8 both promotes apoptosis and inhibits necroptosis through RIPK1 cleavage, its inhibition would be expected to shift cells toward necroptosis if these pathways were active. The lack of response to both inhibitors suggests that cell death at this stage may be driven by alternative mechanisms, and follow-up studies on dual inhibition with zVAD and Nec1 may provide clarity [27,28].

This in vitro model also recapitulates aspects of the biphasic blood–brain barrier (BBB) disruption seen in vivo after TBI. The initial phase of TBI involves direct mechanical damage and necrosis, followed by a delayed, inflammation-driven phase that further compromises barrier integrity [29]. We observed significantly elevated levels of total interleukin-1β (IL-1β), a key pro-inflammatory cytokine, in injured astrocytes (*p* = 0.0066) and microglia (*p* = 0.0162), even in the presence of Nec-1 or zVAD, indicating that early inflammation occurs independently of these programmed cell death pathways.

Parallel analysis of bioactive (cleaved) IL-1β revealed unexpected, cell-type-specific regulation. In astrocytes, CCI + zVAD increased cleaved IL-1β levels compared to injury alone (*p* = 0.0069), suggesting that IL-1β processing may occur after CCI via caspase-independent, serine proteases, cathepsins, or extracellular MMP/serine protease activity and warranting follow-up using selective protease inhibitors and assays for inflammasome assembly [30,31,32]. Microglia, in contrast, showed no significant changes in IL-1β cleavage after injury. In endothelial cells, Nec-1 increased cleaved IL-1β levels (*p* = 0.0029), while pericytes exhibited divergent responses—Nec-1 reduced IL-1β cleavage (*p* = 0.0304), whereas zVAD caused an increase (*p* = 0.0307). These findings highlight complex, cell-specific regulation of inflammatory signaling, where apoptosis and necroptosis inhibitors can have distinct, paradoxical and off-target effects, presenting challenges in the focused study of these pathways [25,33].

Because IL-1β can be released in unprocessed form during necrotic death and in cleaved form during apoptosis, the pattern of IL-1β release and processing helps differentiate the mode of cell death [34]. The elevated total IL-1β levels in astrocytes and microglia, together with the lack of effect from Nec-1 or zVAD, point toward necrotic cell death that does not require caspase or RIPK1 activity.

Further, phosphorylated RIPK1 (pRIPK1), a necroptosis activation marker [35], was undetectable across monocultures, co-cultures, and timepoints (5 min to 7 days) post-CCI, validated with a positive control at each time point (Figure 1C–F; Supplementary Figures S2–S4). While our model of the human NVU suggests a lesser role of necroptosis following TBI, additional studies will be required to clarify the role of necroptosis in early injury responses in human NVU models, which has been reported as a driver in rodent TBI studies [36,37]. Ultimately, in the absence of RIPK1 and caspase-mediated apoptosis, the observed increases in LDH likely reflect non-programmed necrosis or transient membrane disruption rather than canonical necroptosis or apoptosis [38]. Given the mechanical forces imposed by the CCI paradigm, these findings are consistent with shear stress–associated membrane compromise and secondary inflammatory effects as seen by IL-1β modulation [39]. Thus, LDH in this context serves primarily as an indicator of membrane integrity and mechanical stress response, not as a marker of specific cell death pathways.

To further interpret these data, cleaved caspase-1 ELISAs were performed on cell culture media 24 h after CCI in astrocyte, endothelial, and pericyte monocultures, as well as in all co-culture combinations of endothelial cells, astrocytes, pericytes, and microglia (Supplementary Figure S9). In monocultures, astrocytes displayed significant increases in cleaved caspase-1 levels after CCI (*p =* 0.0106), which was further enhanced by Nec-1 treatment (*p = 0*.0088). Pericytes showed similar increases following CCI (*p = 0*.0029) and CCI+Nec-1 (*p =* 0.0031), while endothelial cells showed a significant increase from sham only in the CCI+Nec-1 condition (*p =* 0.0473).

In co-cultures, cleaved caspase-1 levels were largely unchanged in EA (endothelial–astrocyte) cultures. However, EP (endothelial–pericyte) and EM (endothelial–microglia) co-cultures displayed significant increases in CCI+Nec-1 conditions (EP: *p =* 0.006; EM: *p =* 0.0482), with EP cultures also showing increases after CCI alone (*p* = 0.0413). Astrocytes demonstrated enhanced reactivity when co-cultured with pericytes, with significant differences between sham and CCI in AP (*p =* 0.0373) and AM (*p <* 0.0001) cultures, as well as Nec-1 exacerbating CCI effects in AM cultures (*p = 0*.0151). PM cultures showed robust increases from sham following CCI (*p < 0*.0001) and CCI+Nec-1 (*p <* 0.0001).

These data illustrate that CCI induces caspase-1 cleavage in a cell-specific manner, which, coupled with the lack of IL-1β cleavage following CCI, suggests that caspase-1 may act through uncoupled pyroptosis, contributing to LDH release as seen in this model [40]. Uncoupled pyroptosis shifts the role of caspase-1 from an IL-1β cleaving enzyme to primarily a Gasdermin D cleaving enzyme, causing uncoupled pyroptosis and cell lysis, releasing pro-IL-1β into the extracellular space along with LDH, which should not be released under controlled inflammasome and IL-1β processing conditions [39,41]. Consistent with these mechanisms, total IL-1β levels increased in astrocytes and microglia after CCI, but cleaved IL-1β remains unchanged or is decreased in all cell types (Figure 1). Hence, LDH release can be attributed to shear-induced necrosis as a primary driver and caspase-1-mediated uncoupled pyroptosis as a secondary inflammatory signal, bypassing apoptosis, necroptosis, and IL-1β cleavage [40,42]. Ultimately, CCI induced cell-type-specific injury characterized by increased LDH release and IL-1β production, predominantly in astrocytes and microglia, while pericytes were largely resistant. The absence of RIPK1 or apoptosis-dependent effects, together with elevated cleaved caspase-1, indicates that early NVU injury involves mechanical necrosis and caspase-1–mediated uncoupled pyroptosis rather than classical apoptosis or necroptosis.

### 3.2. Cell-Specific Influence on Barrier Integrity Following CCI

**Figure 2:**
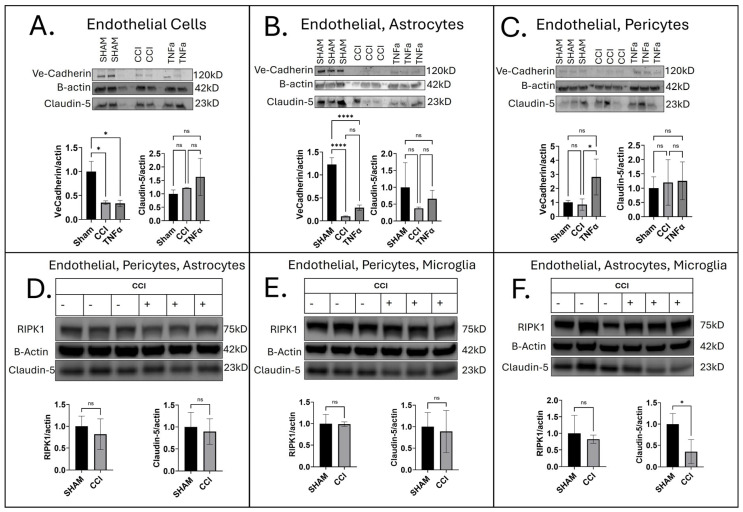
Junctional Markers in Co- and Tricultures. (**A**) Endothelial monoculture, representative Western blots and densitometry calculations 24 h after CCI injury show the expression levels of junctional markers. (**B**,**C**) Endothelial co-culture, representative Western blots and densitometry calculations with astrocytes and pericytes included in the 3D model 24 h after CCI injury show the expression levels of junctional markers. (**D**–**F**) Endothelial triculture, representative Western blots and densitometry calculations with astrocytes, pericytes, and microglia included in the 3D model, 24 h after CCI injury, show the expression levels of junctional markers. Data are presented as the mean ± SEM. *n* = 3 per group. Differences among media released from injured scaffolds were estimated using one-way ANOVA. Comparisons for each pair were carried out by employing Tukey–Kramer. *p* < 0.05 considered significant; ns, not significant (*p* > 0.05); * *p* < 0.05; **** *p* < 0.0001.

To determine how different NVU cell types influence barrier integrity after injury, we analyzed monocultures, co-cultures, and tricultures at 24 h following CCI or treatment with h-TNF-α (10 ng/mL). Endothelial monocultures were compared to co- and triculture conditions in which endothelial cells remained the constant cell type. This design allowed us to assess how varying combinations of neurovascular support cells influence endothelial responses to CCI. By maintaining consistent endothelial seeding across all conditions, any observed differences reflect synergistic or modulatory effects of the added support cells rather than unequal cell loading or baseline variability. In monocultures, endothelial cells alone showed reduced VE-cadherin after both CCI (*p =* 0.0314) and h-TNF-α exposure (*p =* 0.0293), while Claudin-5 expression remained unchanged (Figure 2A). In co-cultures, endothelial–astrocyte pairs displayed a robust loss of VE-cadherin after CCI and TNF-α (*p <* 0.0001), with no change in Claudin-5. In contrast, endothelial–pericyte co-cultures maintained VE-cadherin levels after CCI, though direct TNF-α treatment led to an increase (*p =* 0.0485); Claudin-5 remained stable in both conditions (Figure 2C). In tricultures, the response varied by cellular composition: EPA and EPM cultures showed no significant differences in Claudin-5 or RIPK1 after CCI (Figure 2D,E), whereas EAM cultures exhibited a marked reduction in Claudin-5 following CCI (0.6429 ± 0.2150, *p =* 0.0403) without changes in total RIPK1 (Figure 2F). Together, these results highlight that the impact of injury on endothelial junctional integrity is strongly dependent on the presence and type of supporting NVU cells, with astrocytes exerting the greatest destabilizing influence on VE-cadherin and microglia contributing to Claudin-5 loss in complex multicellular settings. Microglia–astrocyte crosstalk is a key driver of secondary neuronal damage after contusion, and our prior work has identified mitochondrial dysregulation in microglia as a critical mechanism underlying neurodegeneration and neuroinflammation after TBI [22]. Importantly, both cell types play dual roles in the injury response—on one hand promoting reactive and BBB-disruptive processes through cytokine and chemokine release as well as MMP activation, while on the other hand providing supportive, neuroprotective functions that stabilize the barrier and protect the brain [43]. In rodent models, the BBB opens in the first few hours after CCI, reseals, and then is followed by delayed barrier opening in the subsequent 3–7 days after injury [44,45]. In a human in vitro model, the timing of these expression changes (24 h) may be different from a species standpoint and/or culture setup and cell-type requirements. To further elucidate BBB damage mechanisms following TBI in human models, chronic timepoints at and beyond 7 days are necessary for comparison to well-studied rodent models. Our model remains limited by the absence of circulating blood components such as leukocytes, platelets, and plasma proteins, which normally contribute to secondary injury cascades and vascular dysfunction in vivo, leaving room for further model development in future studies. These constraints should be considered when interpreting findings from human-derived systems. These data suggest that endothelial barrier disruption following CCI was highly dependent on NVU composition, with astrocytes exerting the strongest destabilizing effect on VE-cadherin and microglia contributing to Claudin-5 loss in multicellular cultures. These findings demonstrate that BBB integrity after injury is governed by distinct, cell-specific interactions within the NVU rather than by endothelial responses alone.

### 3.3. Paracrine Media Analysis from Injured Monocultures

Paracrine signaling between NVU cell types occurs after TBI [46]. To evaluate these interactions, media were extracted from injured scaffolds and analyzed for cytokine profiles using cytoblots targeting 40 inflammatory cytokines. Signals were quantified and normalized to internal positive controls.

**Figure 3:**
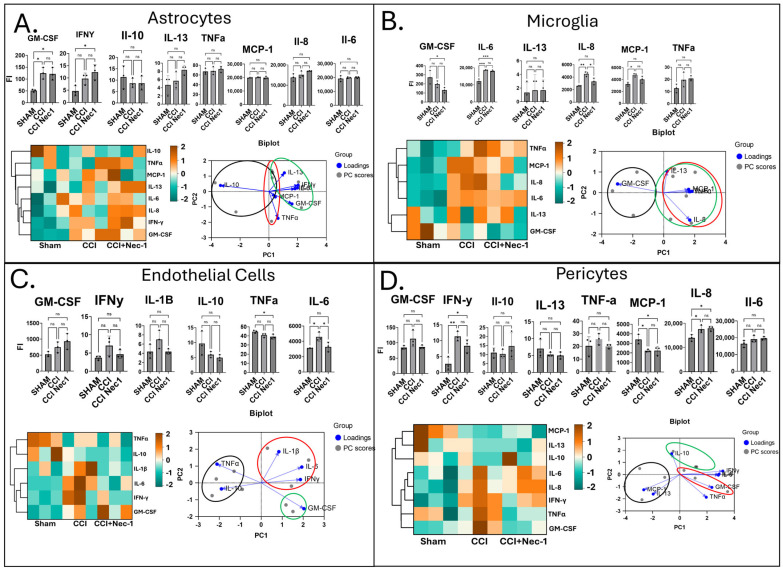
Cytokine Release Characterization in Monocultures after Injury. (**A**–**D**) Cytokines assayed in the media 24 h after CCI trauma in astrocyte alone (**A**), microglia alone (**B**), endothelial cell alone (**C**), and pericyte alone cultures (**D**). Statistically significant cytokines based on ANOVA between individual monoculture cell groups. Cytokine blot intensities were normalized and are reported in a heat map. Heat scores for each individual sample are represented in red, denoting higher relative concentration. and green, denoting lower relative concentration, according to z-scores. Principal component analysis was used to interpret cytokine profiles. A biplot of statistically significant cytokines based on one-way ANOVA shows contributions of cytokines to principal component analysis placements. Circles on PCA plots indicate clustering of sham (black), CCI (red), and CCI+NEC-1 (green) samples for each group. Comparisons for each pair were carried out employing Tukey–Kramer. *p* < 0.05 considered significant; ns, not significant (*p* > 0.05); * *p* < 0.05; ** *p* < 0.01; *** *p* < 0.001.

Differentially expressed cytokines were identified by means of one-way ANOVA across all targets (Figure 3A–D). Heatmaps illustrate distinct release profiles across 3D monocultures of endothelial cells, pericytes, microglia, and astrocytes (Figure 3A–D), with clustering and principal component analyses further resolving cell-type-specific patterns (Figure 3). Endothelial cells and pericytes displayed clear differences between SHAM, CCI, and CCI+Nec1 groups, whereas astrocytes and microglia showed overlapping profiles for CCI and CCI+Nec1. This aligns with earlier data showing no change in cell death or IL-1β release in astrocytes and microglia with Nec-1 treatment, suggesting that glial cell death in this system is unlikely to be strongly necroptosis-dependent. In contrast, cytokine profiles from endothelial cells and pericytes appeared more sensitive to Nec-1 treatment.

Prior rodent studies have implicated astrocytes and microglia as major RIPK1-dependent drivers of neuroinflammation [37,47,48], whereas our findings in human 3D monocultures tentatively suggest a greater role for vascular-associated cells. This discrepancy could reflect species-specific differences, the influence of in vivo systemic immune cells absent in vitro, or off-target effects of Nec-1 that complicate interpretation [25]. Nonetheless, our data are in line with recent human cell culture reports showing that endothelial and perivascular cells mount strong necroptosis-sensitive inflammatory responses [49]. Together, these results highlight the complexity of NVU signaling in TBI and underscore the need for future studies across mouse and human cell types to clarify pathway activation post-CCI. In sum, paracrine cytokine analysis demonstrated that Nec-1 modulated inflammatory profiles predominantly in endothelial and pericyte cultures, revealing a vascular-specific necroptosis sensitivity distinct from the glial response following CCI.

### 3.4. Effects of Paracrine Media from Injured Monocultures on Brain Endothelial Monolayers

**Figure 4:**
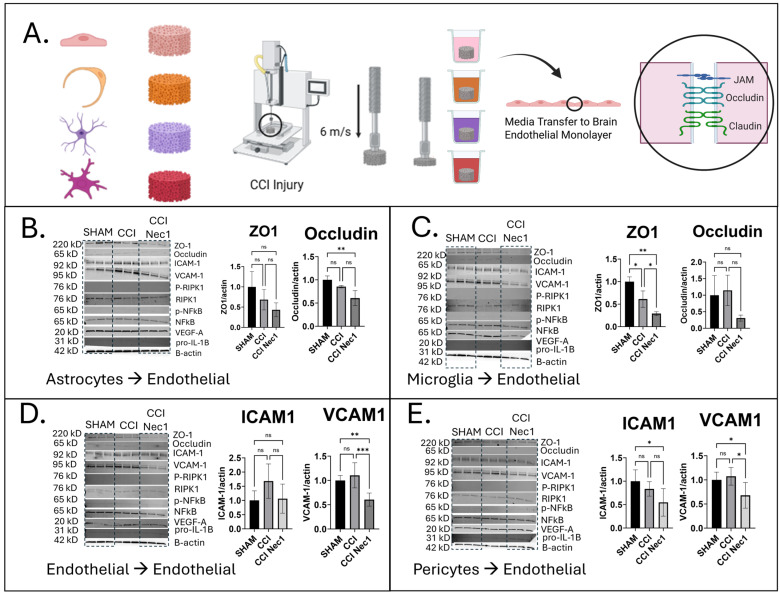
Paracrine Media Transfers Disrupt Endothelial Responses. (**A**) Schematic of seeding primary cells into scaffolds, followed by mechanical trauma with a piston, and then extracting the media after 24 h post-injury and adding onto a confluent endothelial monolayer in 2D. (**B**–**E**) Media from injured astrocytes (**B**), microglia (**C**), endothelial cells (**D**), and pericytes (**E**) were added onto the endothelial monolayer for 72 h and markers of barrier function and inflammation were presented and quantified with densitometry based on one-way ANOVA. Comparisons for each pair were carried out by employing Tukey–Kramer. *p* < 0.05 considered significant; ns, not significant (*p* > 0.05); * *p* < 0.05; ** *p* < 0.01; *** *p* < 0.001. Figure 4A was created with BioRender 2025 (Tufts University institutional license).

While CCI and necroptosis inhibition were analyzed in individual NVU cell types, the impact of support cells on endothelial function remained unclear. To investigate this, we assessed paracrine signaling by transferring conditioned media from injured 3D cultures onto confluent brain endothelial monolayers. After 72 h, endothelial lysates were collected and analyzed for markers of barrier integrity (ZO-1 and Occludin), dysfunction (ICAM-1 and VCAM-1), and inflammation or cell death (pRIPK1, RIPK1, pNFkB, NFkB, VEGF-A, and pro-IL-1β). These results are shown in Figure 4, with additional densitometry and controls in Supplementary Figures S5 and S6.

Notably, ZO-1 protein levels decreased in endothelial cell monolayers post-treatment with conditioned media from CCI microglia cultures (*p =* 0.0202), an effect not rescued by pretreatment with Nec-1 (astrocytes *p =* 0.1045, microglia: *p* = 0.0010) (Figure 4B,C). Occludin levels also decreased after endothelial cells were exposed to conditioned media from CCI astrocyte cultures (*p =* 0.0095), even with Nec-1 pretreatment. VCAM-1 and ICAM-1, markers of endothelial dysfunction and leukocyte recruitment [50], were upregulated in endothelial cells exposed to CCI-conditioned media from endothelial cells, microglia, astrocytes, and pericytes (Figure 4D,E, Supplementary Figures S6 and S7). Interestingly, Nec-1 pretreatment reduced VCAM-1 expression compared with CCI alone in endothelial cells treated with conditioned media from CCI cultures of endothelial cells (*p =* 0.0009) and pericytes (*p =* 0.0199), effects not seen with Nec-1 treatment alone (Supplement Figure S7). In endothelial cells and pericytes, cytokine release profiles and endothelial marker expression after paracrine media transfers were different in the presence of Nec-1 compared to CCI. There may be some indication based on these differences that RIPK1 activity might still be an important signaling pathway for these cells’ responses in vitro and worthy of exploring alternate phosphosites (Ser166 is the canonical marker of activation). RIPK1 kinase activity is canonically indicated by phospho-RIPK1 at Ser166 for Western blotting and ELISA studies. Additional phosphosites are linked to RIPK1 kinase activity, including Ser161, which, if studied, may provide more clarity on these conflicting data [51]. However, we suspect that the most likely explanation for differences is from the off-target effects of Nec-1 on indoleamine 2,3-dioxygenase (IDO). Necrostatin-1 inhibits IDO-mediated inflammation in addition to RIPK1 kinase activity, which makes it difficult for researchers to define the exact role of RIPK1 activation when this inhibitor is used [25].

Three-dimensional CCI models help distinguish whether NVU-endothelial signaling is mediated by paracrine factors or direct cell–cell contact. In this study, phospho-RIPK1 was not detected after CCI in endothelial monocultures, co-cultures, or tricultures. Because contusion injuries in vivo introduce blood products into the parenchyma [52], we next tested whether hemin, a breakdown product of red blood cells, [53] could activate RIPK1 signaling. We found that hemin (100 µM) exposure caused toxicity in monocultures, co-cultures, and tricultures; however, no induction of pRIPK1 or reduction in Claudin-5 expression was observed at 24 h. This result suggests that while hemin that may enter the brain after contusion is toxic, these pathways are not necroptosis dependent and do not contribute to further dysregulation of tight junctions.

These findings suggest conditioned media from injured glial and vascular monocultures disrupted endothelial tight-junction integrity and upregulated adhesion molecules, with Nec-1 selectively mitigating VCAM-1 induction but not junctional loss, suggesting partial, pathway-specific necroptosis mediated paracrine inflammation following CCI.

## 4. Conclusions

Developing a better understanding of how cell–cell interactions modulate the function of the NVU and the integrity of brain endothelial junctions is important to gain insight into TBI signaling and downstream neurodegeneration [54,55]. Using our model, we demonstrate both direct contact and paracrine pathways of barrier disruption after CCI injury. Endothelial monocultures and endothelial–astrocyte co-cultures exhibited reduced VE-cadherin expression, whereas pericytes conferred protection against TBI-induced barrier disruption. In tricultures with astrocytes and microglia, Claudin-5 levels decreased, highlighting the complexity of multicellular responses. Paracrine studies further revealed that injured microglia impaired endothelial ZO-1 expression, while culture media from injured astrocytes, pericytes, and endothelial cells upregulated VCAM-1. Despite these functional changes, no pRIPK1 activation was detected, and Nec-1 treatment failed to prevent cell death within 24 h, suggesting that necroptosis does not drive early injury responses in these 3D TBI models. As an in vitro modeling platform intended to investigate cell-specific contributions following TBI, there remain limitations. Complementary viability assays (e.g., annexin V/PI, TUNEL, p-MLKL, and AlamarBlue) were not included, which would help distinguish apoptotic, necrotic, and pyroptotic. Collectively, these findings position this human 3D NVU model as a useful platform for dissecting cell-specific and paracrine mechanisms that underlie BBB disruption after TBI. While there is ample space for further mechanistic investigation, this model demonstrates that vascular and glial cells contribute to endothelial barrier dysfunction through non-programmed necrosis and possibly uncoupled pyroptosis-linked signaling. Overall, this study supports a more active role for the NVU in modulating TBI-induced inflammation and barrier compromise. Future applications of this system may facilitate preclinical screening of targeted interventions under human-relevant conditions.

## Data Availability

The original contributions presented in this study are included in the article/Supplementary Material. Further inquiries can be directed to the corresponding authors.

## Supplementary Materials

The following supporting information can be downloaded at https://www.mdpi.com/article/10.3390/jfb16120454/s1. Figure S1: Endothelial cell, astrocyte, and pericyte colonization of 3D silk–collagen scaffolds. Figure S2: Cell-specific pRIPK1 (pSer166) activity in mono- and co-cultures. Figure S3: Cell-specific pRIPK1 activity for each NVU cell type. Figure S4: Cell-specific loading controls for each NVU cell type. Figure S5: Cell-specific tight junction and neuroinflammatory protein analysis. Figure S6: Paracrine media and protein transfers to assess the role of NVU cell–endothelial cell communication on junctional and inflammatory targets. Figure S7: Nec-1 controls of paracrine protein transfers to assess the role of NVU cell–endothelial cell communication on junctional and inflammatory targets. Figure S8: Relative expression of junctional markers in pericytes and endothelial cells. Figure S9: Cleaved caspase-1 expression is increased in mono- and co-cultures.
