# Supplementary material for: Three-Dimensional Human Neurovascular Unit Modeling Reveals Cell-Specific Mechanisms of Traumatic Brain Injury
Source: J Funct Biomater. 2025 Dec 7;16(12):454. doi: 10.3390/jfb16120454 (PMC12733534; doi:10.3390/jfb16120454)
Supplement: Supplementary file 1 [file jfb-16-00454-s001.zip › jfb-3921736-supplementary.pdf]

Supplementary materials:

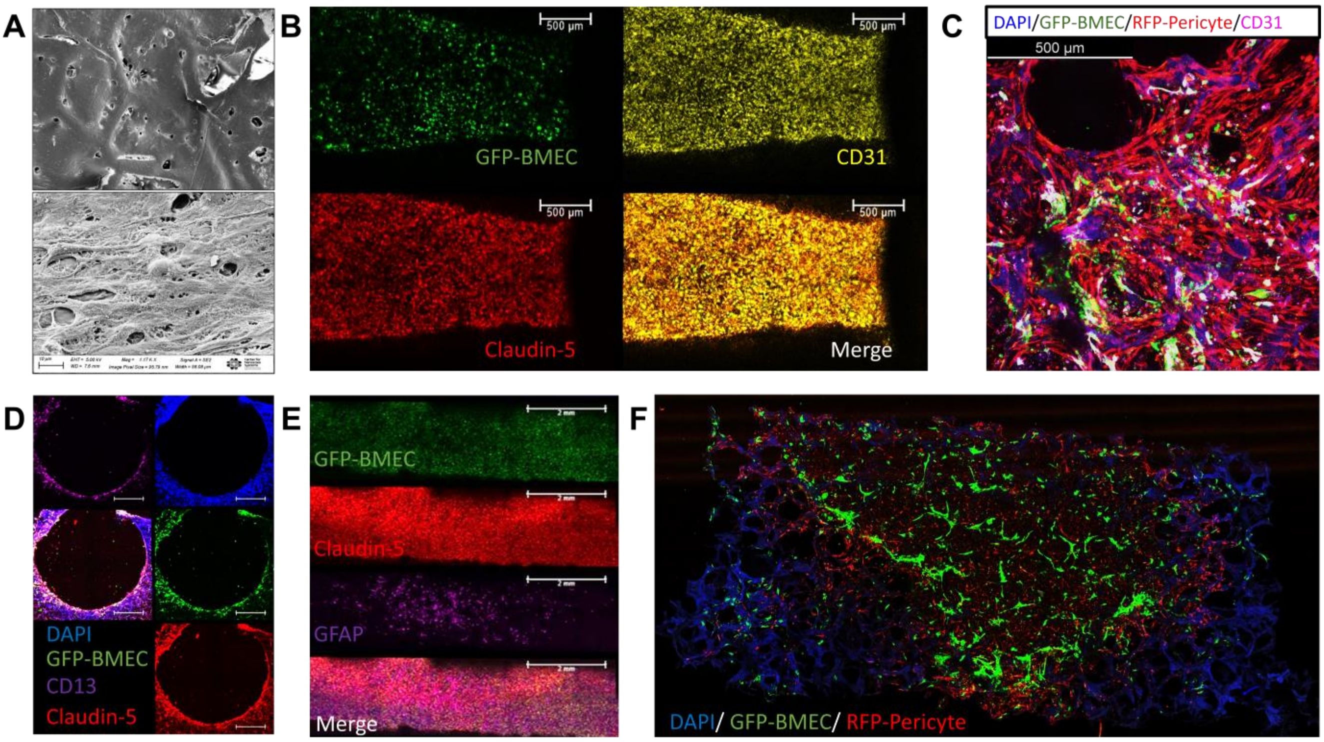

Figure S1. Endothelial cell, astrocyte, and pericyte colonization of 3D silk-collagen scaffolds. (**A**) Representative one week SEM micrographs illustrating endothelial cell, pericyte, and astrocyte scaffold colonization (bottom) compared to the control, empty scaffold (top). Representative confocal micrographs illustrating cell specific marker expression within 3D scaffolds for (**B**) 4 day endothelial only, (**C**) 7 day endothelial and pericyte, (**D**) 4 day endothelial and pericyte, (**E**) 4 day endothelial and astrocyte, and (**F**) 7 day endothelial and pericyte cultures. Scale bars, (**A**) 10µm, (**B-D**) 500µm, (**E**) 2mm, (F) 1mm.

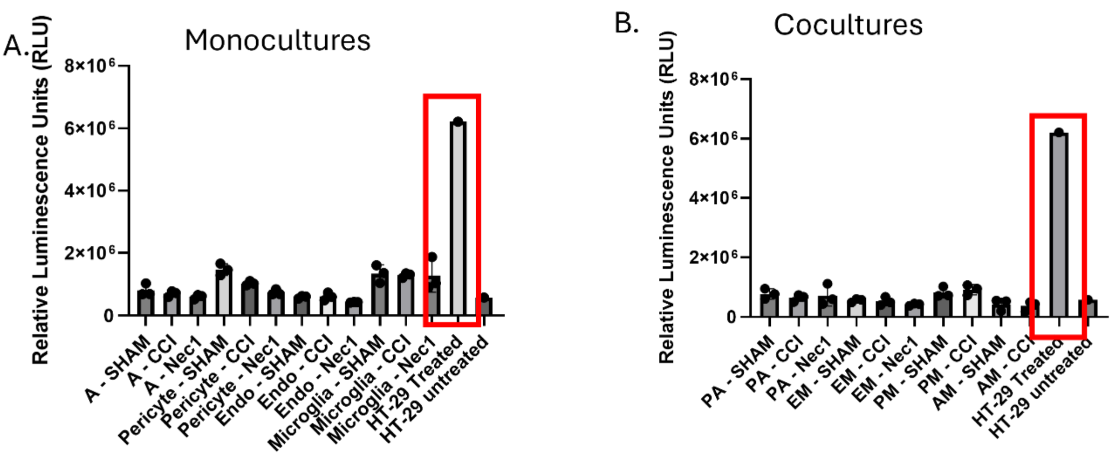

Figure S2. Cell-specific pRIPK1 (pSer166) Activity in Mono and Cocultures. (**A**) Relative expression of pRIPK1 activity measured by ELISA in monocultures for astrocytes, pericytes, endothelial cells, and microglia with either sham, CCI or CCI+NEC-1 insults. (**B**) Relative expression of pRIPK1 activity measured by

ELISA for cocultures with either sham, CCI or CCI+NEC-1 insults, n=3 per group. Negative and Positive controls were established using untreated HT-29 cells or HT-29 cells treated with TNFa, SM-164, zVAD to activate pRIPK1 (outlined in red).

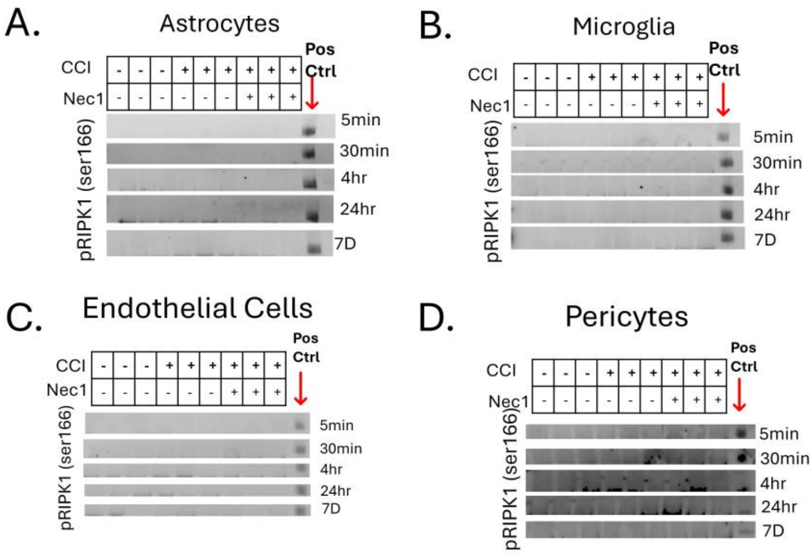

Figure S3. Cell-specific pRIPK1 Activity for each NVU cell type. (**A**) Astrocytes, (**B**), Microglia, (**C**) Endothelial cells, and (**D**) pericytes probed for pRIPK1 (ser166) by western blot at 5 minutes, 30 minutes, 4, hours, 24 hours, or 7 days. Sham, CCI, NEC-1, and CCI+NEC-1 groups were analyzed, *n*=3 per group. Recombinant pRIPK1 was used as a positive control at each timepoint.

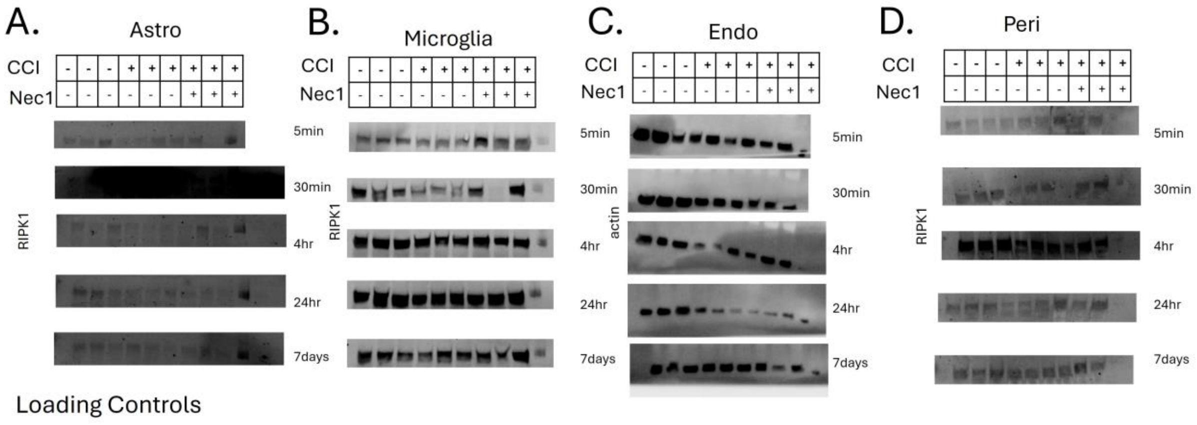

Figure S4. Cell-specific loading controls for each NVU cell type. (**A**) Astrocytes, (**B**), Microglia, (**C**) Endothelial cells, and (**D**) pericytes probed for total RIPK1 or beta actin by western blot at 5 minutes, 30 minutes, 4, hours, 24 hours, or 7 days. Sham, CCI, NEC-1, and CCI+NEC-1 groups were analyzed, *n*=3 per group. Recombinant RIPK1 was used to ensure antibody specificity.

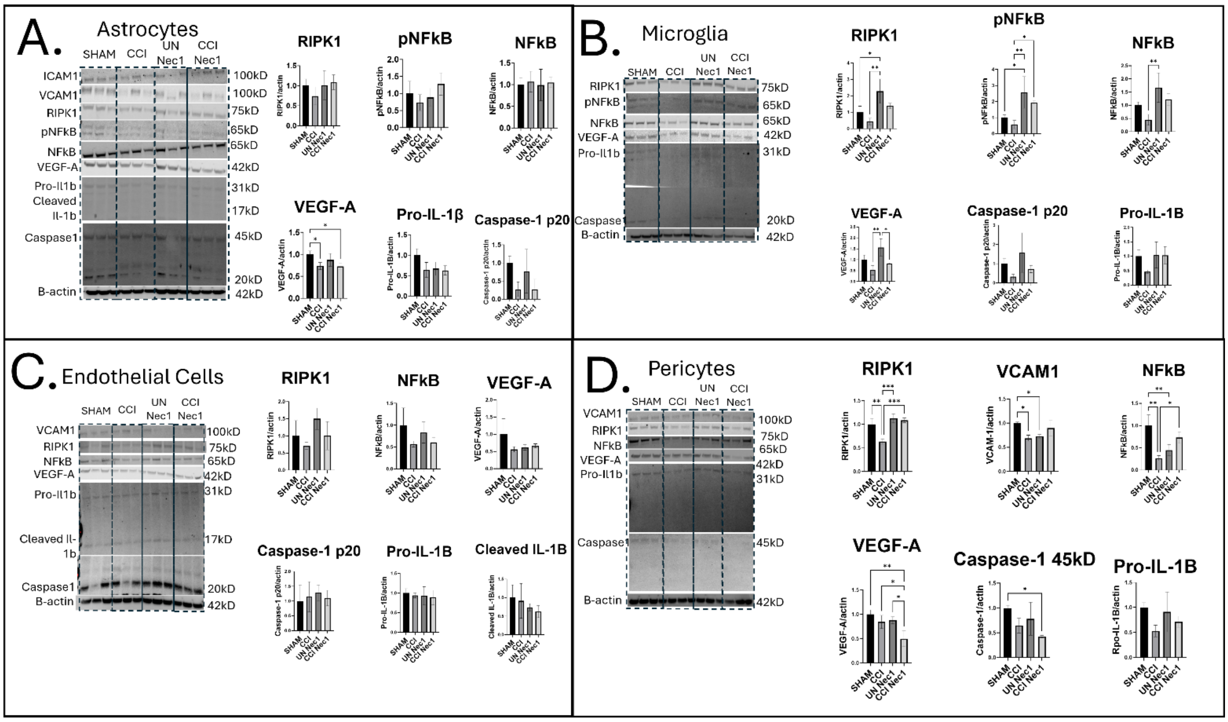

Figure S5. Cell-specific tight junction and neuroinflammatory protein analysis. (A) Astrocytes, (B), Microglia, (C) Endothelial cells, and (D) pericytes probed for tight junction and neuroinflammatory markers by western blot 24 hours after either sham, CCI, NEC-1, and CCI+NEC-1 groups, presented and quantified with densitometry based on one-way ANOVA. Comparisons for each pair were carried out employing Tukey-Krammer. $p<0.05$ considered significant; *$p<0.05$, **, $p<0.01$,***, $p<0.001$, ****, $p<0.0001$, $n = 3$ per group.

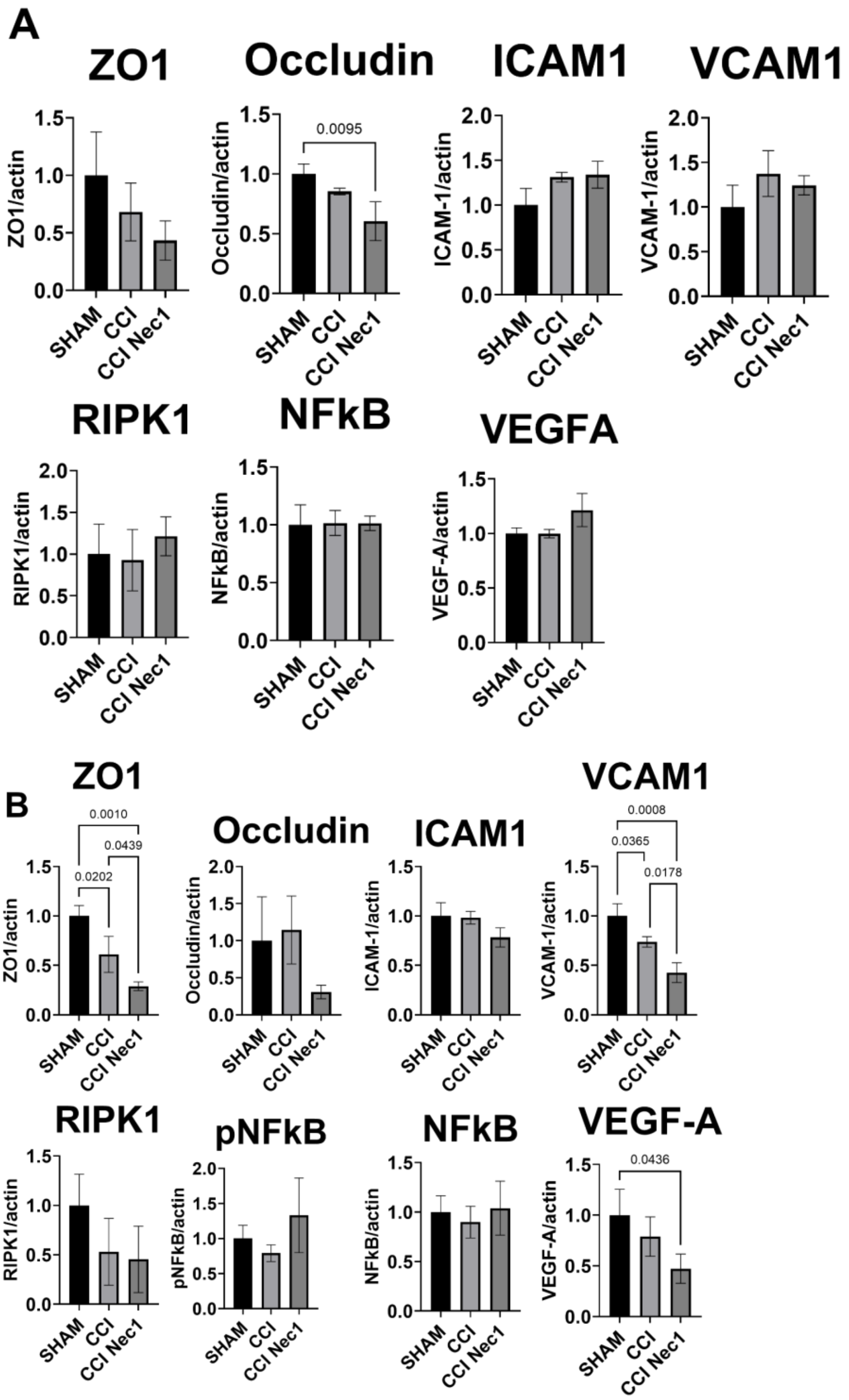
A
ZO1
Occludin
ICAM1
VCAM1
ZO1/actin
Occludin/actin
0.0095
ICAM-1/actin
VCAM-1/actin
RIPK1
NFkB
VEGFA
RIPK1/actin
NFkB/actin
VEGF-A/actin
SHAM
CCI
CCI Nec1
B
ZO1
0.0010
0.0439
0.0202
ZO1/actin
Occludin
Occludin/actin
ICAM1
ICAM-1/actin
VCAM1
0.0008
0.0365
0.0178
VCAM-1/actin
RIPK1
RIPK1/actin
pNFkB
pNFkB/actin
NFkB
NFkB/actin
VEGF-A
0.0436
VEGF-A/actin

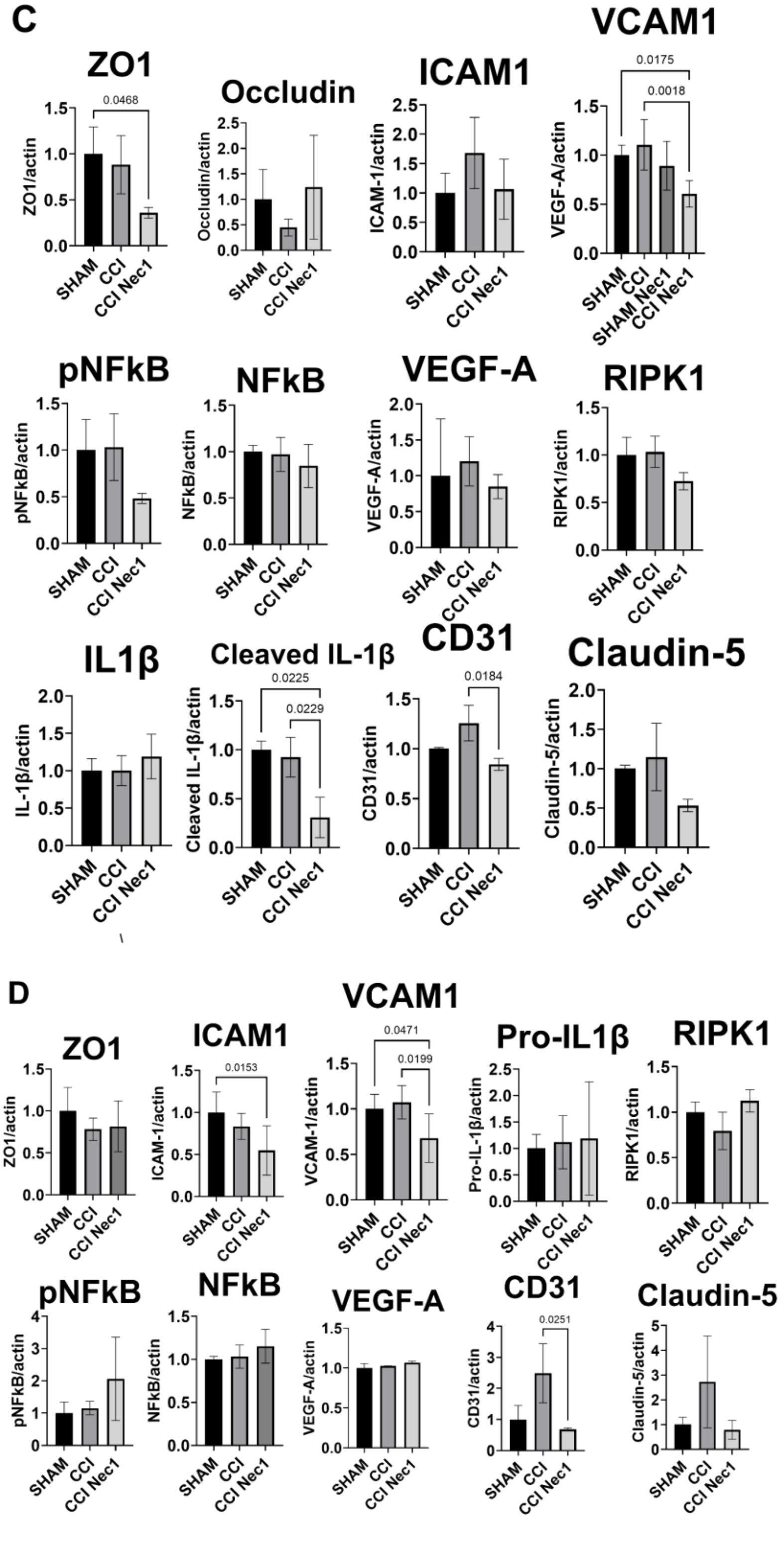

Figure S6. Paracrine media and protein transfers to assess the role of NVU cell-endothelial cell communication on junctional and inflammatory targets. (**A**) Astrocyte to endothelial cell media transfer, (**B**), microglia to endothelial cell transfer, (**C**) endothelial cell to endothelial cell protein transfer, and (**D**) pericyte to endothelial cell protein transfer were used to assess endothelial cell tight junction and neuroinflammatory markers by western blot 24 hours after either sham, CCI, and CCI+NEC-1 groups, presented and quantified with densitometry based on one-way ANOVA. Comparisons for each pair were

carried out employing Tukey-Krammer. p<0.05 considered significant; *p<0.05, **,p<0.01,***, p<0.001, ****, p<0.0001, *n*=3 per group.

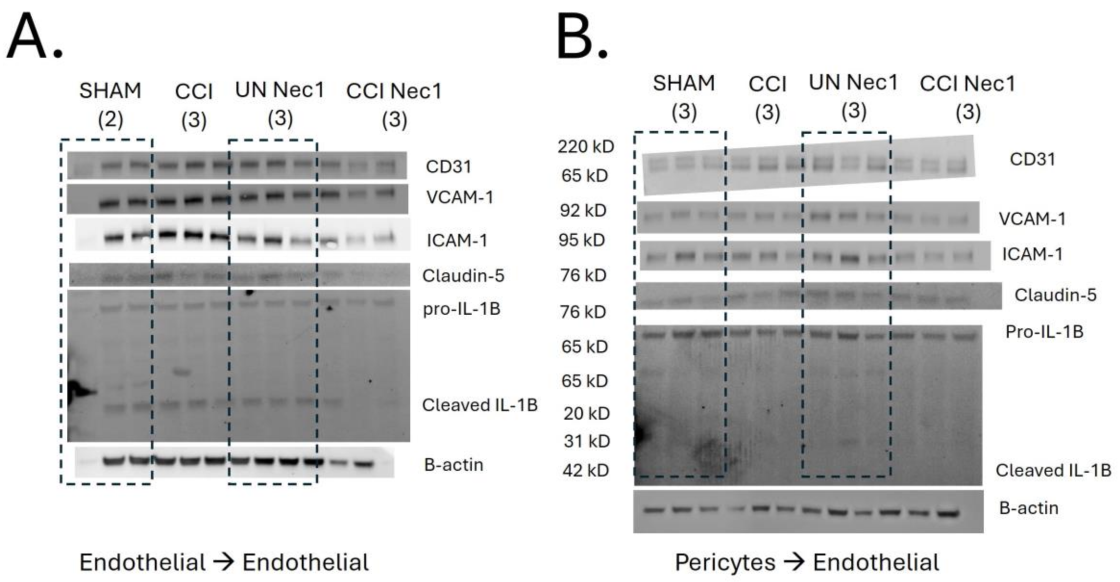

Figure S7. Nec-1 controls of paracrine protein transfers to assess the role of NVU cell-endothelial cell communication on junctional and inflammatory targets. Nec-1 controls for (**A**) Endothelial cell to endothelial cell protein transfer, and (**B**) pericyte to endothelial cell protein transfer were used to assess endothelial cell tight junction and neuroinflammatory markers by western blot 24 hours after either sham, CCI, Nec-1, and CCI+NEC-1 groups, *n*=3 per group.

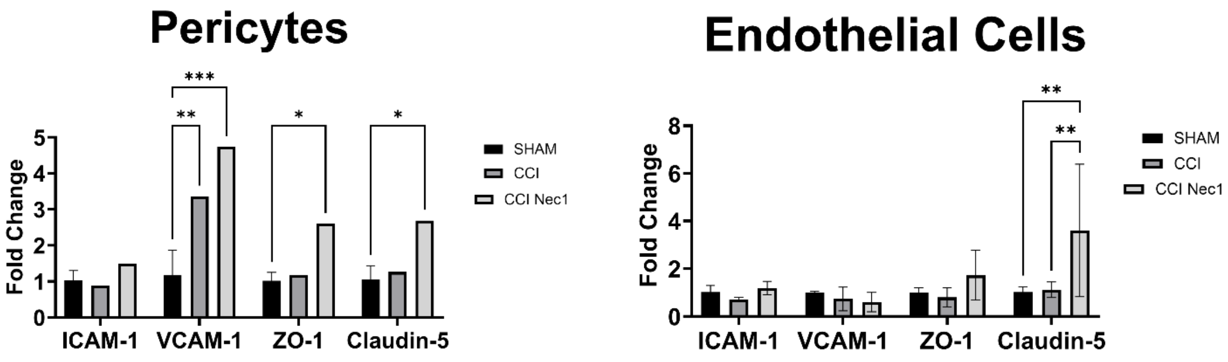

Figure S8. Relative expression of junctional markers in pericytes and endothelial cells. qPCR using cell lysates (**A**) Pericytes and (**B**), endothelial cells to assess tight junctional markers for either sham, CCI, or CCI+NEC-1 groups, presented and quantified fold change based on one-way ANOVA. Comparisons for each pair were carried out employing Tukey-Krammer. p<0.05 considered significant; *p<0.05, **,p<0.01,***, p<0.001, ****, p<0.0001, *n*=3 per group.

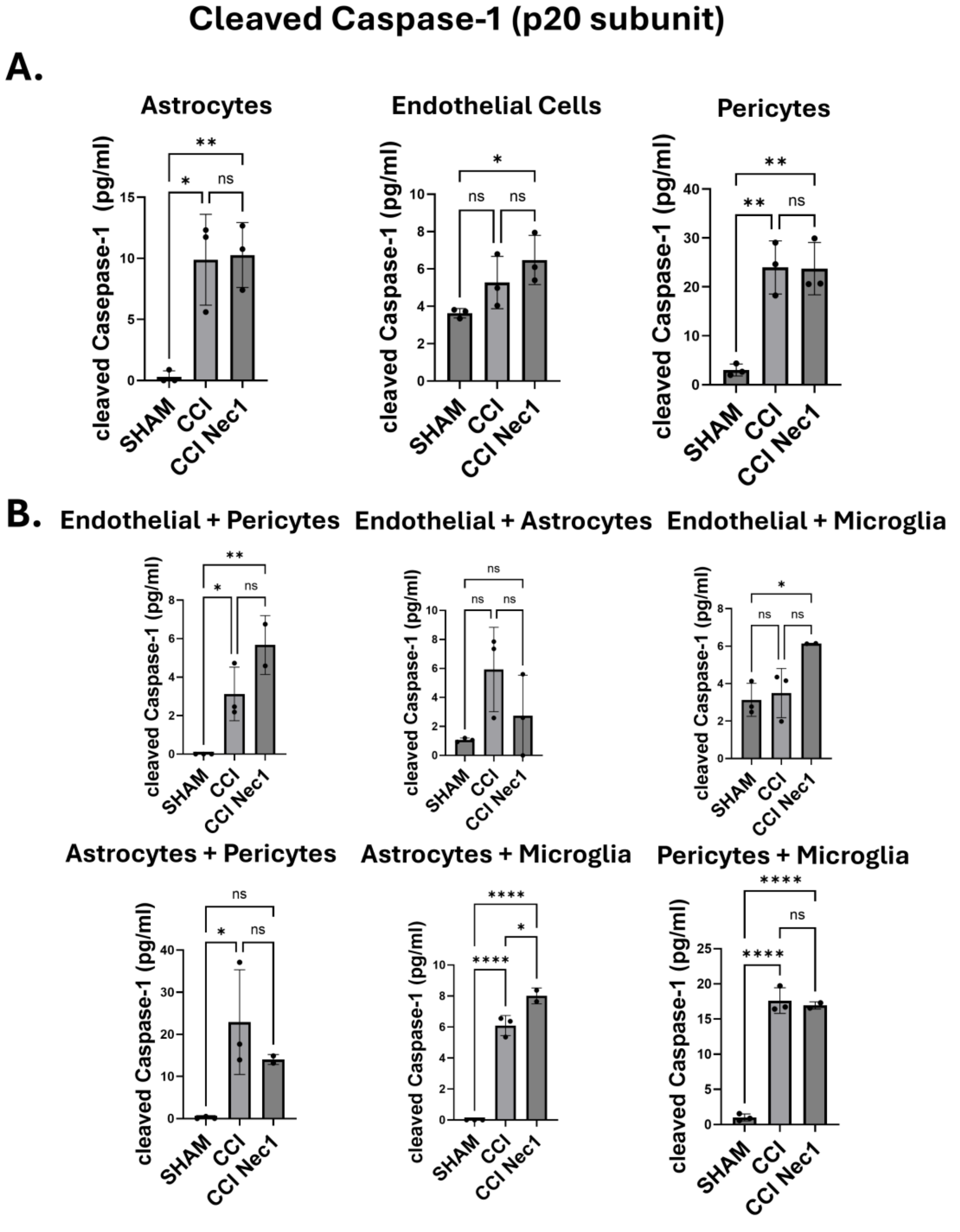

Figure S9. Cleaved caspase-1 expression is increased in mono- and co-cultures. Cleaved caspase-1 detected in media by ELISA 24hrs following (**A**) CCI in 3D monocultures of astrocytes, endothelial cells, and pericytes and (**B**) CCI in co-cultures of pericytes, astrocytes, microglia, and brain endothelial cells., presented and quantified as pg/ml secreted, based on one-way ANOVA. Comparisons for each pair were carried out employing Tukey-Krammer. p<0.05 considered significant; *p<0.05, **,p<0.01,***, p<0.001, ****, p<0.0001, *n*=3 per group.
